# Genetic analysis of single-minded 1 gene in early-onset severely obese children and adolescents

**DOI:** 10.1371/journal.pone.0177222

**Published:** 2017-05-04

**Authors:** Daniela Stanikova, Marek Buzga, Patrik Krumpolec, Martina Skopkova, Martina Surova, Barbara Ukropcova, Lubica Ticha, Miroslava Petrasova, Dominika Gabcova, Miroslava Huckova, Lucie Piskorova, Jan Bozensky, Marian Mokan, Jozef Ukropec, Ivona Zavacka, Iwar Klimes, Juraj Stanik, Daniela Gasperikova

**Affiliations:** 1 Laboratory of Diabetes and Metabolic Disorders, Institute of Experimental Endocrinology, Biomedical Research Center, Slovak Academy of Sciences, Bratislava, Slovakia; 2 Department of Pediatrics, Medical Faculty of Comenius University, Bratislava, Slovakia; 3 Institute of Social Medicine, Occupational Health and Public Health, University of Leipzig, Leipzig, Germany; 4 Department of Physiology and Pathophysiology, Faculty of Medicine, University of Ostrava, Ostrava, Czech Republic; 5 Institute of Pathophysiolgy, Faculty of Medicine, Comenius University, Bratislava, Slovakia; 6 Department of Pediatrics, Medical Faculty of Safarik University, Kosice, Slovakia; 7 Department of Pediatrics, Vitkovice Hospital, Ostrava, Czech Republic; 8 Department of Internal Medicine, Jessenius Medical Faculty of Comenius University, Martin, Slovakia; 9 Department of Biomedical Sciences, Faculty of Medicine, University of Ostrava, Ostrava, Czech Republic; 10 Center for Pediatric Research Leipzig, University Hospital for Children & Adolescents, University of Leipzig, Leipzig, Germany; University of Oslo, NORWAY

## Abstract

**Background:**

Inactivating mutations of the hypothalamic transcription factor singleminded1 (SIM1) have been shown as a cause of early-onset severe obesity. However, to date, the contribution of *SIM1* mutations to the obesity phenotype has only been studied in a few populations. In this study, we screened the functional regions of *SIM1* in severely obese children of Slovak and Moravian descent to determine if genetic variants within *SIM1* may influence the development of obesity in these populations.

**Methods:**

The *SIM1* promoter region, exons and exon-intron boundaries were sequenced in 126 unrelated obese children and adolescents (2–18 years of age) and 41 adult lean controls of Slovak and Moravian origin. Inclusion criteria for the children and adolescents were a body mass index standard deviation score higher than 2 SD for an appropriate age and sex, and obesity onset at less than 5 years of age. The clinical phenotypes of the *SIM1* variant carriers were compared with clinical phenotypes of 4 *MC4R* variant carriers and with 27 unrelated *SIM1* and *MC4R* mutation negative obese controls that were matched for age and gender.

**Results:**

Seven previously described *SIM1* variants and one novel heterozygous variant p.D134N were identified. The novel variant was predicted to be pathogenic by 7 *in silico* software analyses and is located at a highly conserved position of the SIM1 protein. The p.D134N variant was found in an 18 year old female proband (BMI 44.2kg/m^2^; +7.5 SD), and in 3 obese family members. Regardless of early onset severe obesity, the proband and her brother (age 16 years) did not fulfill the criteria of metabolic syndrome. Moreover, the variant carriers had significantly lower preferences for high sugar (*p* = 0.02) and low fat, low carbohydrate, high protein (*p* = 0.02) foods compared to the obese controls.

**Conclusions:**

We have identified a novel *SIM1* variant, p.D134N, in 4 obese individuals from a single pedigree which is also associated with lower preference for certain foods.

## Introduction

Single minded 1 (SIM1) is a bHLH/PAS (basic helix-loop-helix/Per-Arnt-Sim) transcription factor that forms a functional dimer with another bHLH/PAS family member, ARNT2 [1]. SIM1 plays a key role in neuronal differentiation of nucleus paraventricularis in the hypothalamus [2] which is important for regulation of energy homeostasis by interacting with pathways influencing melanocortin signaling. SIM1 acts downstream of the melanocortin 4 receptor (MC4R) [3], and mutations in this gene are a major cause of monogenic obesity in humans. Mice models showed that *SIM1* haploinsufficient mice develop obesity and have higher stature likely due to early life hyperphagia, while *SIM1* overexpression decreases food intake in mice [4]. Similarly in humans, *SIM1* mutations seem to be involved in the pathogenesis of obesity; for example, a balanced translocation disrupting the *SIM1* gene was found in a girl with severe non-syndromic early-onset obesity [5], and patients with 6q deletions encompassing the *SIM1* gene present with Prader-Willi-like phenotypes including obesity and developmental delay [6]. Recently, it was shown that point mutations in *SIM1* cause monogenic obesity as well [7,8]. Similar to *MC4R* mutation carriers, *SIM1* mutation carriers also have early-onset obesity caused by increased appetite and food seeking behavior persisting into adulthood [7,9]. Additional features include increased respiratory quotient, hyperinsulinemia, and signs of neurobehavioral abnormalities, as well as, emotional lability, autistic features or learning disabilities (concentration and memory disorders) [7,10]. Patients could also have Prader-Willi-like phenotypes including delayed psychomotor development or intellectual disability and dysmorphic features [8,11]. Moreover, several studies suggest that *SIM1* polymorphisms may also contribute to polygenic obesity [12,13]. Nevertheless, the contribution of *SIM1* variants to early-onset obesity have so far only been studied in a few populations [7,8,10].

The aim of our study was to search for *SIM1* mutations in children with early-onset severe obesity; and, to describe detailed metabolic phenotypes of the mutation carriers and compare their phenotypes with the phenotypes for obese individuals not carrying *SIM1* variant.

## Patients and methods

### Children and adolescent cohort

One hundred and twenty-six children (68 girls and 58 boys) of Slovak (n = 113) and Moravian (n = 13) origin age 2 to 18 years with body mass index (BMI) standard deviation scores > 2 SD for an appropriate age and sex and obesity onset at less than 5 years of age were recruited by pediatric endocrinologists throughout Slovakia during the years 2009–2014, and in the Ostrava region of Moravia during the years 2011–2014. All patients with other causes of obesity (e.g., pharmacological and previously confirmed syndromic obesity) were excluded. Anthropometric data (height and weight) were measured by specialized nurses in the pediatric endocrinology outpatient clinics. The BMI percentiles and standard deviation score were calculated using local anthropometric references [14]. The mean BMI standard deviation score of the children included in the study was 5.1±2.4 SD and mean age at obesity onset was 2.5±1.5 years (range 0.4 – 5years). Twenty-six of the probands had at least one of the following additional features: (i) developmental delay, (ii) intellectual disability, and (iii) learning or behavioral disabilities. One patient had facial dysmorphic features characteristic for Prader-Willi-like syndrome. During regular health check-ups, information on genetic testing was given and blood samples were drawn for DNA isolation. Clinical data for the proband and family relatives and genealogical history were provided by the referring physician. Samples of venous blood (8 ml) were collected into EDTA tubes (Sarstedt, Nümbrecht, Germany) for DNA analysis and were transported to the laboratory with the informed consent form and patient’s medical history questionnaire.

### Lean controls

A control population of 41 adult lean subjects of Slovak origin with BMI < 25kg/m2 (mean 22.0 ± 2.2) was examined for variants in the *SIM1* gene.

### Obese controls

Obese controls were used for the phenotype comparison with individuals carrying the novel p.D134N *SIM1* variant. This cohort consisted of 14 unrelated *SIM1* and *MC4R* mutation negative adult obese individuals (age 31–59 years, BMI 34.1 ± 4.9 kg/m2), 13 unrelated *SIM1* and *MC4R* mutation negative peri- and postpubertal children and adolescents (7 females age 15.8 ± 2.7 years and BMI SDS +4.4 ± 1.6; 6 males age 15.4 ± 2.3 years and BMI SDS +5.4 ± 1.7).)

### Molecular genetic analyses

Genomic DNA was extracted from peripheral leukocytes using standard procedures, and the promoter, all exons, and intron-exon boundaries of the *SIM1* gene were amplified by polymerase chain reaction (PCR) using previously described primers [15]. PCR products were sequenced using standard methods on an ABI/Hitachi 3500 resp. 3130 (Applied Biosystems, Warrington, UK) and were compared with the reference sequence NM_005068.2 using SeqScape software (version 2.1.1; Applied Biosystems, Warrington, UK). Novelty of the identified missense mutations was verified using public variation databases: dbSNP147 (www.ncbi.nlm.nih.gov/SNP), EVS (evs.gs.washington.edu/EVS/ [accessed 25.10.2016]) and ExAC (exac.broadinstitute.org [accessed 25.10.2016]) DNA sequence conservation was assessed using GERP, phyloP100way, phastCons100way, and multiz100way provided by UCSC Genome Browser (http://genome.ucsc.edu) [16–18]. Variant pathogenicity was tested using the following *in silico* analyses: SIFT [19], PROVEAN (http://provean.jcvi.org) [20], PolyPhen-2 (http://genetics.bwh.harvard.edu/pph2/index.shtml), MutationAssesor (http://mutationassessor.org) [21], Mutation Taster (http://www.mutationtaster.org) [22], CADD (http://cadd.gs.washington.edu/score) [23], and SNPs&GO (http://snps.biofold.org) [24]. Sequence alignment of SIM proteins was done by Clustal Omega at www.uniprot.org.

### Hormonal and other biochemical analyses

Glucose, lipids, alanine transaminase, aspartate transaminase, thyrotropin, thyroxine, and insulin levels were measured from frozen plasma using standard laboratory protocols in the Department of Clinical Biochemistry of the Children University Hospital in Bratislava, Slovakia. Glycosylated hemoglobin (HbA1c) was evaluated from whole blood using a LPLC DiaSTAT analyzer (Bio-Rad). All the values were transformed to the Diabetes Control and Complications Trial (DCCT) percentage and International Federation of Clinical Chemistry (IFCC) values (http://www.ngsp.org/ifccngsp.asp) [25,26]. Fasting measures and oral glucose tolerance tests (oGTT) with 75g glucose were performed after 3 days on a high-saccharide diet and normal physical activity followed by a 12-hour overnight fast according to the standardized WHO protocol [27].

### Metabolic parameters

Metabolic syndrome in adolescents and adults was evaluated using the criteria recommended by the International Diabetes Federation [28]. Selected insulin secretion and insulin resistance indices were assessed from the fasting values and oGTTs using previously published formulas: fasting insulin (INS0) [29], homeostatic model assessment—insulin resistance (HOMA-IR) [30], peak insulin level during an oGTT (INSMAX) [31], area under the curve for insulin (AUCINS), ratio of areas under the curve for insulin and glucose levels during an oGTT (AUCINS/AUCGLU) [32], and whole body insulin sensitivity index (Matsuda WBISI) [33]. Where appropriate, glucose and insulin concentrations were transformed from mmol/L and nmol/L to mg/dL and ng/mL using coefficients of 0.05551 for glucose and 6.945 for insulin, respectively.

### Indirect calorimetry and food preference questionnaire

Basal metabolic rate and metabolic substrate preference (respiratory quotient, RQ) were calculated from concentrations of oxygen (VO2) and carbon dioxide (VCO2) in exhaled air (RQ = VCO2/VO2, REE = [3.9 (VO2) + 1.1 (VCO2)] 1.44 –Weir equation). Indirect calorimetry measurements (n = 3) were performed in the fasted state just before the oGTT. Thirty-minute breath-by-breath measurements were initiated after a 30-minute bed rest at thermal comfort conditions using the Ergostik (Geratherm Respiratory, Germany). Results were compared with indirect calorimetry data of the *MC4R* mutation carriers and family members related to *MC4R* and *SIM1* mutation carriers [34]. The *MC4R* mutation carriers were selected for the comparison because of their similar phenotypes to *SIM1* mutation carriers [7]. Food Preference Questionnaire assesses preferences for 72 foods [35]. Participants rate each food hedonically on a 9-point scale by rating how much they like each food, with 1 = dislike extremely, and 9 = like extremely. Major energy source content in preferred food was evaluated, and individual preference was characterized in context of (i) high fat and high simple sugar score; (ii) high fat and high complex carbohydrate score; (iii) high fat, low carbohydrate and high protein score; (iv) low fat and high simple sugar score; (v) low fat and high complex carbohydrate score; (vi) low fat, low carbohydrate and high protein score; (vii) high sugar score; (viii) high complex carbohydrate score; (ix) high protein score and (x) fat preference score.

### Psychological examination

Testing for neurobehavioral abnormalities, such as mental retardation, emotional instability, learning disability, behavioral disorders, and autistic features was performed by TMT—Trail Making Test, Rawen progressive matrices, and by the method of numbers repeating [36].

### Statistics

Comparisons betweeneby t-test for numeric variables and by Fisher exact test for nominal variables. P values less than 0.05 were considered as statistically significant in all analyses. Statistical analyses were performed using SPSS (version 17; SPSS, Chicago, IL, USA).

### Ethic committee approval

The present study was approved by the National Institute of Endocrinology and Diabetology in Lubochna, Slovakia and the Ethics Committee of Faculty of Medicine of University of Ostrava, Czech Republic. Study participants or the parents of minors who participated in the study signed a written informed consent for the genotype and phenotype analyses.

## Results

### *SIM1* genotypes of the obese children

**Fig 1:**
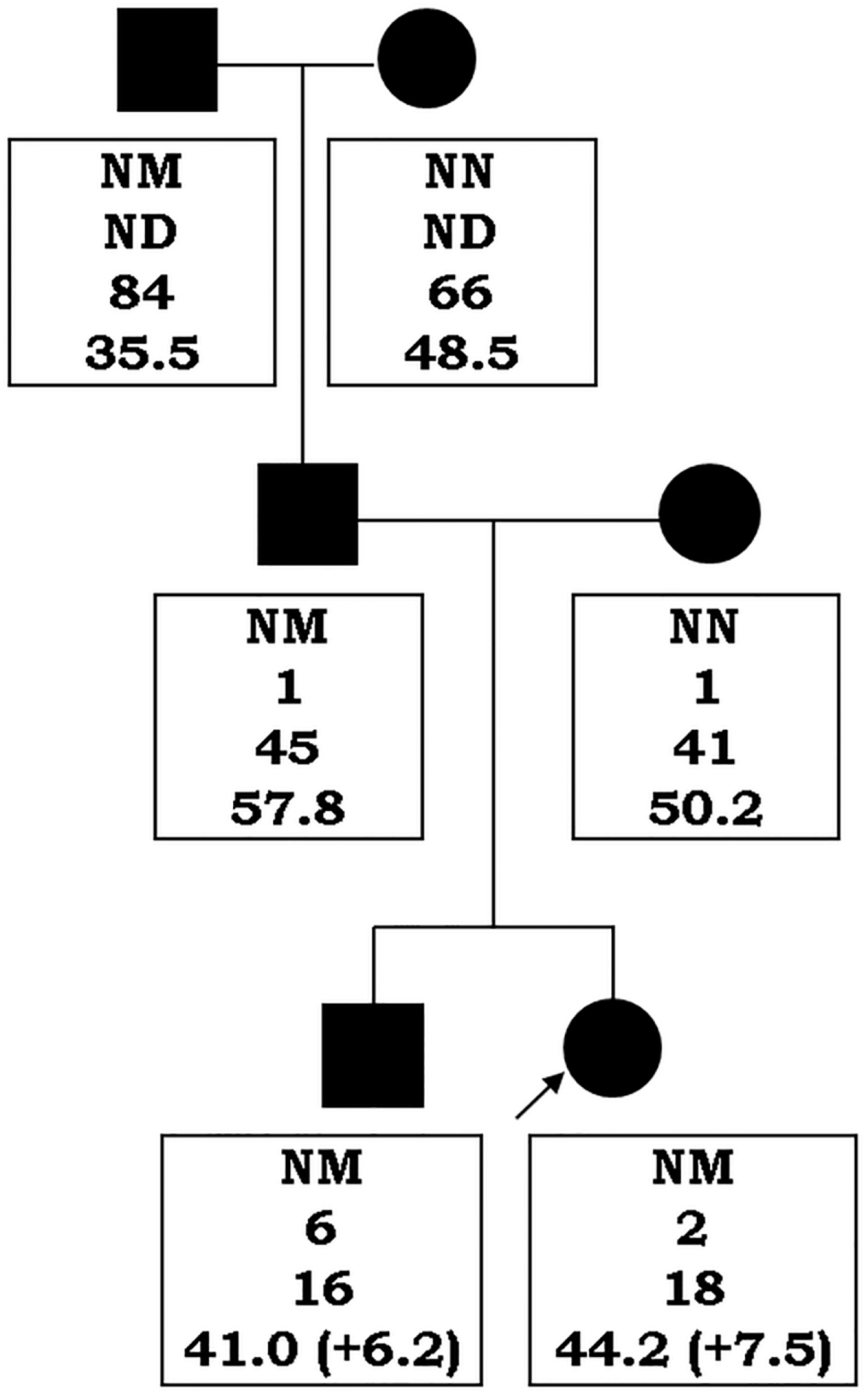
Pedigree of the family with the novel *SIM1* variant p.D134N. Squares represent males; circles represent females; filled symbols indicate obese individuals. Proband is indicated by an arrow. The text below each individual indicates mutational status (NM—heterozygous p.D134N carrier; NN—non-carrier), age at diagnosis of obesity, current age, and BMI (BMI SDS). ND—not determined.

The *SIM1* promoter and exons were sequenced in 126 unrelated obese children and adolescents (2–18 years of age) and 41 adult lean controls of Slovak and Moravian origin. In one obese proband, a novel heterozygous *SIM1* variant c.400G>A (p.D134N) was identified. The same heterozygous genotype was also found in the proband’s brother, father, and paternal grandfather; other obese family members, mother and paternal grandmother, were not carriers of the p.D134N variant (Fig 1).

Moreover, seven previously described *SIM1* polymorphisms were found among patients and controls (two nonsynonymous variants in the coding region, c.1054C>A (p.P352T) and c.1112C>T (p.A371V), one synonymous variant, c.1959C>T (p.T653 =), one promotor variant, c.-256G>A, one variant in the 5'UTR, c.-127T>C, as well as two variants in the 3'UTR, c.*113A>T and c.*348A>T) (Table 1).

**Table 1:** Distribution of *SIM1* variants among carriers and *in silico* analyses.

Nucleotide change NM_005068.2	Amino acid change	Variant type	dbSNP	Obese probands (n = 126)	Non-obese adults (n = 41)	Protein domain of SIM1	Allele frequency			GERP++ RS	phyloP 100Way	Phast Cons 100way	Multiz 100way	SIFT prediction: score	polyPhen-2 (HumVar) prediction:score	PROVEAN prediction:score	Mutation assessor functional impact:score	CADD	SNP&GO:disease probability	MutationTaster
							TGP	EVS	ExAC											
**c.(-256)G>A**	-	promotor	rs77109140	1 (0.8%)	0	-	0.02	-	-	-3.7	-1.04	0	0.35	-	-	-	-	9.6	-	Polymorphism
**c.-127T>C**	-	5'UTR	rs41315244	10 (7.9%)	3 (7.3%)	-	0.02	-	-	-2.21	0.16	0.05	0.38	-	-	-	-	7.2	-	Polymorphism
**c.400 G>A**	**p. D134N**	missense	N/A	1 (0.8%)	0	PAS1	-	-	-	5.26	7.73	1	0.88	Damaging:0.037	Possibly dmaging::0.846	Deleterious:-4.27	Low:1.82	34.0	Disease:0.688	Disease causing
**c.1054C>A**	**p.P352T**	missense	rs3734354	32 (25.4%)	14 (34.1%)	C-term	0.18	0.10	0.17	5.8	6.0	1	0.76	Damaging:0.006	Benign:0.052	Neutral:-0.8	Low:1.59	23.9	Neutral:0.115	Polymorphism
**c.1112C>T**	**p.A371V**	missense	rs3734355	33 (25.4%)	15 (34.1%)	NLS	0.18	0.10	0.17	2.86	1.39	0.94	0.78	Tolerated:0.562	Benign:0.003	Neutral:-0.39	Neutral:0.55	19.9	Neutral:0.063	Polymorphism
**c.1959C>T**	**p.T653 =**	silent	rs41318041	5 (4.0%)	0	C-term	0.01	0.02	0.01	-9.51	-1.89	0	0.73	-	-	-	-	1.5	-	Disease causing
c.*113A>T	-	3'UTR	rs41318039	8 (6.3%)	N/A	-	0.01	-	-	-1.13	0.58	0.02	0.59	-	-	-	-	10.2	-	Disease causing
c.*348A>T	-	3'UTR	rs1395119	76 (60.3%)	N/A	-	0.40	-	-	-3.76	-0.74	0	0.55	-	-	-	-	0.03	-	Polymorphism

**TGP—1000 Genomes Project; EVS—Exome Variant Server**—NHLBI **GO Exome Sequencing Project; ExAC—Exome Aggregation Consortium; Cut offs used for in silico prediction tools: SIFT:<0.05, polyPhen-2:>0.8, PROVEAN:<-2.5, SNP&GO:>0.5;**

The missense variant p.D134N has not previously been reported. It is not present in the 1000 Genomes Project phase 3 release included in dbSNP147, nor in the more than 6500 individuals screened in NHLBI GO Exome Sequencing Project, nor in the ExAC database summarizing data of more than 60,000 exomes (Table 1). The aspartic acid at position 134 is located in the PAS1 domain of the protein (S1A Fig). This region is evolutionary conserved at the DNA level (high GERP, phyloP100way, and PhastCons100way scores) as well as at the protein level (Multiz100way) (Table 1). Specifically, the c.400G nucleotide is highly conserved in all species of the UCSC Multiz Alingment of 100 vertebrates and the aspartic acid is replaced by another negatively charged amino acid, glutamic acid, in only one of the 100 vertebrate species in the alignment (S1B Fig). The substitution with asparagine was predicted to be deleterious by SIFT, polyPhen, PROVEAN, MutationAssessor, SNPs&GO, MutationTaster and the CADD score value 34 places this substitution among the 0.04% most deleterious variants in the human genome (Table 1).

### Phenotypes of the p.D134N carriers

The novel SIM1 variant p.D134N was found in one obese female proband born to unrelated parents with normal birth weight and length. She developed obesity at the age of 2 years and she has been continuously gaining weight. She had a learning disability and concentration problems during childhood (reported by her mother). At the time of the examination she was 18 years old and had diffuse obesity with BMI of 44.2 kg/m^2^ (BMI SDS +7.5 SD). She had no dysmorphic features, no specific comorbidities, and normal mental status according to the psychological examination (average intellect level, no concentration or learning disabilities, or emotional lability). She had displayed mild fasting hypertriglyceridemia (1.7 mmol/l); while, fasting and 2hr glycemia during a glucose tolerance test and HDL-cholesterol levels were in the normal range (Table 2). Alanine transaminase, aspartate transaminase, thyrotropin, and thyroxine levels were also in the normal range (data not shown). She did not meet the criteria for metabolic syndrome as defined by the International Diabetes Federation [28].

**Table 2:** Genotype and phenotype characteristics in the proband with the novel p.D134N variant and her family members.

	Genotype for the SIM1 p.D134N variant	Age at examination/Age at obesity onset (yrs)	Dysmorphic features	Learning or behavioral disorders, emotional lability	Height in cm (SDS)	BMI in kg/m^2^ (SDS if ≤ 18 years of age)	Blood pressure (mmHg)	Fasting plasma glucose (mmol/l)	120-minute glucose during an oGTT (mmol/l)	Triglycerides (mmol/l)	HDL-cholesterol (mmol/l)	Fasting plasma insulin (pmol/l)	120-minute insulin during an oGTT (pmol/l)	HbA1c in % (DCCT)/mmol/mol	Metabolic syndrome
**Proband**	**NM**	18/2	no	learning problems in childhood	156 (-1.5)	44.2 (7.5)	126/83	4.6	6.9	**1.7**	1.6	76.4	928.6	5.1/32.2	no
**Brother**	**NM**	16/6	no	no	175 (-0.5)	41.0 (6.2)	133/73	4.1	5.7	1.5	1	**146.5**	786.9	5/31.1	no
**Father**	**NM**	45/1	no	no	171 (-1.3)	57.8	110/68*	**5.5**	**10.4**	**1.8**	**0.9**	**159**	621.6	**6.7/49.7**	**yes**
**Father´s father**	**NM**	84/ND	no	N/A	172 (-1.1)	35.5	N/A	**5.8**	N/A	0.7	1.9	96.5	N/A	N/A	no
**Mother**	NN	41/1	no	no	164 (-0.2)	50.2	108/66	4.6	4.8	**2.1**	**0.8**	**34**	473	**5.8/39.9**	**yes**
**Father’s mother**	NN	66/ND	No	N/A	152 (-2.2)	48.5	N/A	**18.4**	N/A	1.1	**0.9**	117.4	N/A	N/A	**yes**

*value on antihypertensive therapy, ND—not determined, oGTT—oral glucose-tolerance test. Not physiological values are in bold. Symbols for genotype: NM—heterozygous p.D134N variant carrier, NN—p.D134N non-carrier.

The proband’s brother was obese (BMI 41 kg/m2, BMI SDS +6.2 SD, age 16 years) with fasting hyperinsulinemia (146.5 pmol/l); while, his fasting and 2hr oGTT glycemia, lipid profile and blood pressure were in the normal range. The proband’s father was obese (BMI 57.8 kg/m2) and had diabetes mellitus according to the HbA1c value (6.7%; 49.7 mmol/mol), hyperglycemia at 2hr oGTT (10.4 mmol/l), dyslipidemia (triglycerides 1.8 mmol/l and HDL-cholesterol 0.9 mmol/l), and hypertension on treatment. The proband’s paternal grandfather was obese (BMI 35.5 kg/m2) with fasting hyperglycemia (5.8 mmol/l), while other metabolic parameters were in the normal range (Table 2). Despite displaying early onset severe obesity, the proband and her brother did not fulfill the criteria of metabolic syndrome as defined by the International Diabetes Federation [28]. Proband’s father was the only variant carrier who met the criteria for the metabolic syndrome (Table 2).

### Phenotype comparison of p.D134N carriers with obese controls

**Fig 2:**
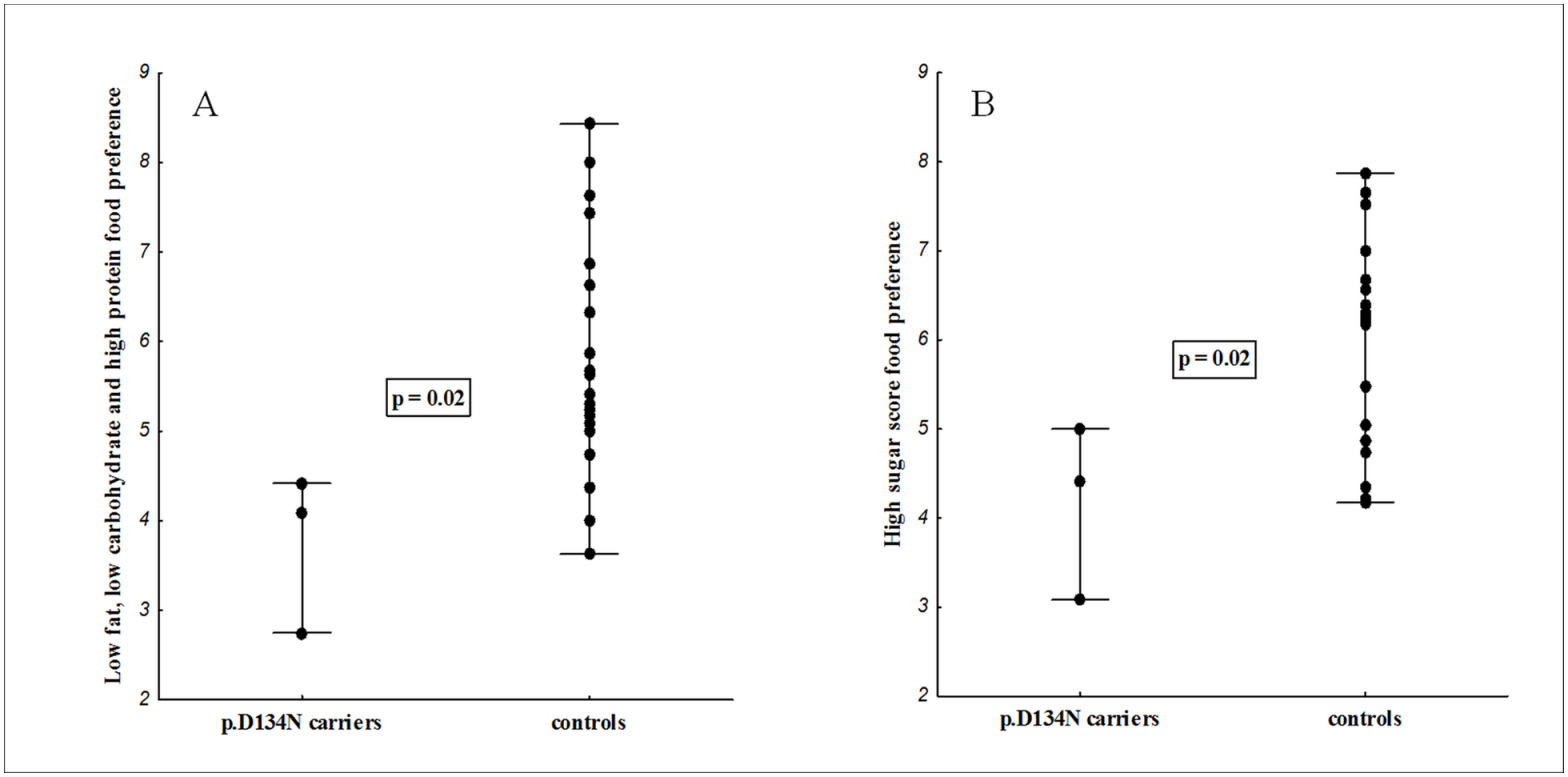
Food preference for the novel SIM1 variant p.D134N carriers (proband, her father and brother) compared to unrelated obese adult controls. A) Food preference for the low fat, low carbohydrate and high protein food and B) Food preference for the high sugar food.

The anthropometric and metabolic phenotypes of p.D134N variant carriers (proband, proband’s brother, and proband’s father) were compared with gender matched, similarly aged unrelated obese controls negative for *SIM1* and *MC4R* mutations (S2 Fig). BMIs for the 2 siblings carrying p.D134N were comparable to the obese controls, while the father’s BMI was higher compared to the controls (S2A and S2B Fig). Height of the variant carriers was apparently lower than in the controls (S2C and S2D Fig), however, the non-carrier family members also had short stature (Table 2). Most of the metabolic phenotypes for the p.D134N carriers were within the range of the obese controls with the possible exception of HDL cholesterol levels (S2H Fig). Moreover, the variant carriers had significantly lower preference for low fat, low carbohydrate and high protein food (*p* = 0.02) and lower preference for high sugar food (*p* = 0.02) but normal preference for other energy substrates compared to the obese controls (Fig 2). Differences in other food preferences were not significant (data not shown).

There were no significant differences in respiratory quotient and resting energy expenditure of the p.D134N variant carriers compared to the *MC4R* mutation carriers or obese family relatives (S3 Fig).

## Discussion

We have sequenced the *SIM1* promoter region and exons in 126 unrelated obese children and adolescents, and 41 adult lean controls from Slovakia and Moravia. One novel *SIM1* variant (p.D134N) in one proband out of 126 (0.79%) obese children with early-onset severe obesity was identified. There are additional immediate family members who are also obese (BMI > 35.5 kg/m^2^) including 3 p.D134N variant carriers and 2 non-carriers. Pathogenicity of the novel variant was supported by *in silico* analyses. Despite several phenotype similarities with the obese controls, p.D134N variant carriers had significantly lower preference for low fat, low carbohydrate and high protein food and lower preference for high sugar food. Moreover, both younger mutation carriers (<20 years) had no other features of metabolic syndrome beside early-onset obesity with high BMI SDS (>6), borderline triglycerides in the proband, and hyperinsulinemia in the brother.

### SIM1 variants causing monogenic obesity

Loss-of-function variants linked to early-onset obesity were previously identified in various regions of the *SIM1* gene [7,8,10], particularly in the N-terminal transactivation domain and the PAS1 and PAS2 domains [7]. Discrepancies between *in silico* and *in vitro* functional analyses were described for several mutations [8]. Furthermore, several loss-of-function mutations confirmed by both *in silico* and *in vitro* functional analyses were found in obese and lean individuals (e.g. p.D707H) [7].

In our case, 7 *in silico* analyses predicted the p.D134N variant to be deleterious. The aspartic acid at position 134 is evolutionary conserved not only in SIM1 (S1B Fig), but also in SIM2 proteins and in the single-minded protein *of Drosophila melanogaster* (S1C Fig). It is located in the PAS1 domain of the protein. The PAS1 and PAS2 domains of bHLH/PAS proteins are responsible for dimerization, stabilizing the DNA binding conformation of the dimer, and assembly of functional transcription complexes [37]. Moreover, a heterozygous mutation p.M136K, in the same domain (PAS1) as the p.D134N variant, caused obesity in mice [38].

In the case of our novel *SIM1* p.D134N variant, functional analyses, which would confirm its impact on SIM1 function, were not performed. This is a major limitation of our study. Nevertheless, all of the mutation carriers were obese, that is at least in agreement with the hypothesis of p.D134N deleteriousness.

### Comparison to other studies

The inclusion criteria in our study were selected according to the main phenotype features of previously published *SIM1* mutation carriers (i.e. severe, early-onset obesity) [7,8]. Moreover, as a part of the *SIM1* mutation carriers has additional symptoms [8], also 26 patients with severe early onset obesity and learning disabilities or dysmorphic features of Prader-Willi-like syndrome were included.

Carriers of the p.D134N variant had childhood onset obesity (≤6 years), significantly increased BMIs (>35 kg/m2), and normal basal metabolic rates according to sex, age and body composition. Compared to Ramachandrappa et al., who showed higher respiratory quotient in *SIM1* mutation carriers compared to obese controls [7], our data showed no significant differences in basal metabolic rate or respiratory quotient for the p.D134N variant carriers compared to the *MC4R* mutation carriers or obese family relatives.

None of the previous studies reported the appearance of metabolic syndrome among the *SIM1* mutation carriers [7,8,10]. In our studied family, metabolic syndrome was diagnosed only in one of four mutation carriers, but in both obese non-carriers (Table 2); and, also in the majority of unrelated obese controls (the differences were not significant). Nevertheless, because of the small number of p.D134N variant carriers, no conclusion on metabolic health could be made. Since *SIM1* is linked to *MC4R* [39,40], the phenotypes of *SIM1* and *MC4R* mutation carriers are similar [7]. According to other studies from Europe, several *MC4R* mutation carriers had the same or even better metabolic health compared to the similarily aged obese non-carriers [41–43]. Conversely, a study in Pima Indians showed an earlier onset of type 2 diabetes in *MC4R* mutation carriers [44]. In a mice model, female *MC4R* mutation carriers were normoglycemic but males were hyperglycemic [45]. Moreover, mice carrying a heterozygous p.M136K mutation of *SIM1* had significantly higher hyperinsulinemia, but the serum glucose levels were not significantly changed compared to wild-type mice [38]. Nevertheless, in the study by Ramachandrappa et al., human *SIM1* and *MC4R* mutation carriers had lower blood pressure and lower heart rate compared to obese controls [7]. Since hypertension is one of the most frequent components of metabolic syndrome, its absence could also influence the prevalence of whole metabolic syndrome in *SIM1* and *MC4R* mutation carriers. Moreover, we have shown that p.D134N variant carriers had significantly lower preference for high sugar food and low fat, low carbohydrate and high protein food compared to the obese controls. Interestingly, changes in food preference (lower preference for high fat diet) were also observed by Xi et al. in SIM1 neuron ablated mice [46].

### Genotype–phenotype interactions

To date, only a few of the described heterogeneous nonsynonymous loss-of function *SIM1* variants fulfilled all of the criteria required for a monogenic obesity mutation (i.e. cosegregation with the obesity phenotype in the family, similar phenotype in all of the mutation carriers, and decreased transcriptional activity of the mutated SIM1 in *in vitro* studies) [7,8]. On the other hand, several loss-of-function variants with decreased *in vitro* activity were found in families with obesogenic environment (i.e. both parents of the proband with a mutation were obese; e.g., two families with p.T46R [8], or p.D707H [7], or families with p.A517V [10], p.R171H, p.P692L, and p.R550H [7]). Similarly, the p.D134N variant was found in a family where all of the members were obese. This could be another limitation of our study as no further genetic analysis, beside sequencing of both the *MC4R* and *SIM1* genes (with negative results), was performed in the family. Nevertheless, there are some differences between carriers and non-carriers; for example, better metabolic health (3 of 4 mutation carriers did not fulfill the criteria for metabolic syndrome) and lower preference for high sugar food and low fat, low carbohydrate and high protein food in variant carriers compared to the obese controls.

### Implication for clinicians

Although *SIM1* gene mutations are a rare cause of obesity, their clinical importance is based on the high risk of morbid obesity (frequently with BMI >50kg/m2). Therefore, an early diagnosis with focused prevention and therapy seems to be reasonable.

## Conclusions

We have identified a novel heterozygous nonsynonymous *SIM1* variant (p.D134N) in four family relatives showing phenotype differences in metabolic health and changed food preference compared to related and unrelated obese non-carriers.

## Supporting information

S1 Fig Location of the p.D134N variant in the SIM1 protein. A) The p.D134N is located in the PAS1 domain involved in heterodimerization of SIM1 with ARNT2 transcription factor; B) the aspartic acid at the position 134 is conserved in 99 of 100 vertebrate species as shown in the caption from the UCSC Genome Browser (only one representative species from 8 different vertebrate subsets is displayed); C) The aspartic acid at the position 134 is also conserved in other SIM proteins down to *Drosophila melanogaster*.
(PDF)

S2 Fig Selected anthropometric and metabolic parameters of the *SIM1* variant p.D134N carriers compared to controls. Abbreviations: PC—sex and age matched obese controls for proband, BC—sex and age matched obese controls for proband’s brother, FC—sex and age matched obese controls for proband’s father. The plots display the original values and ranges (for controls). The p-values for differences in unpaired t-test were displayed only if p<0.05.
(PDF)

S3 Fig Indirect calorimetry data of the *SIM1* variant p.D134N carriers compared to *MC4R* mutation carriers and obese family relatives negative for *MC4R* and *SIM1* loss of function mutations (negative controls). Data (n = 3) are shown as means ± SE.
(PDF)
