# Supplementary figures and images for: Genetic analysis of single-minded 1 gene in early-onset severely obese children and adolescents
Source: PLoS One. 2017 May 4;12(5):e0177222. doi: 10.1371/journal.pone.0177222 (PMC5417716; doi:10.1371/journal.pone.0177222)

S1 Fig. Location of the variant p.D134N.

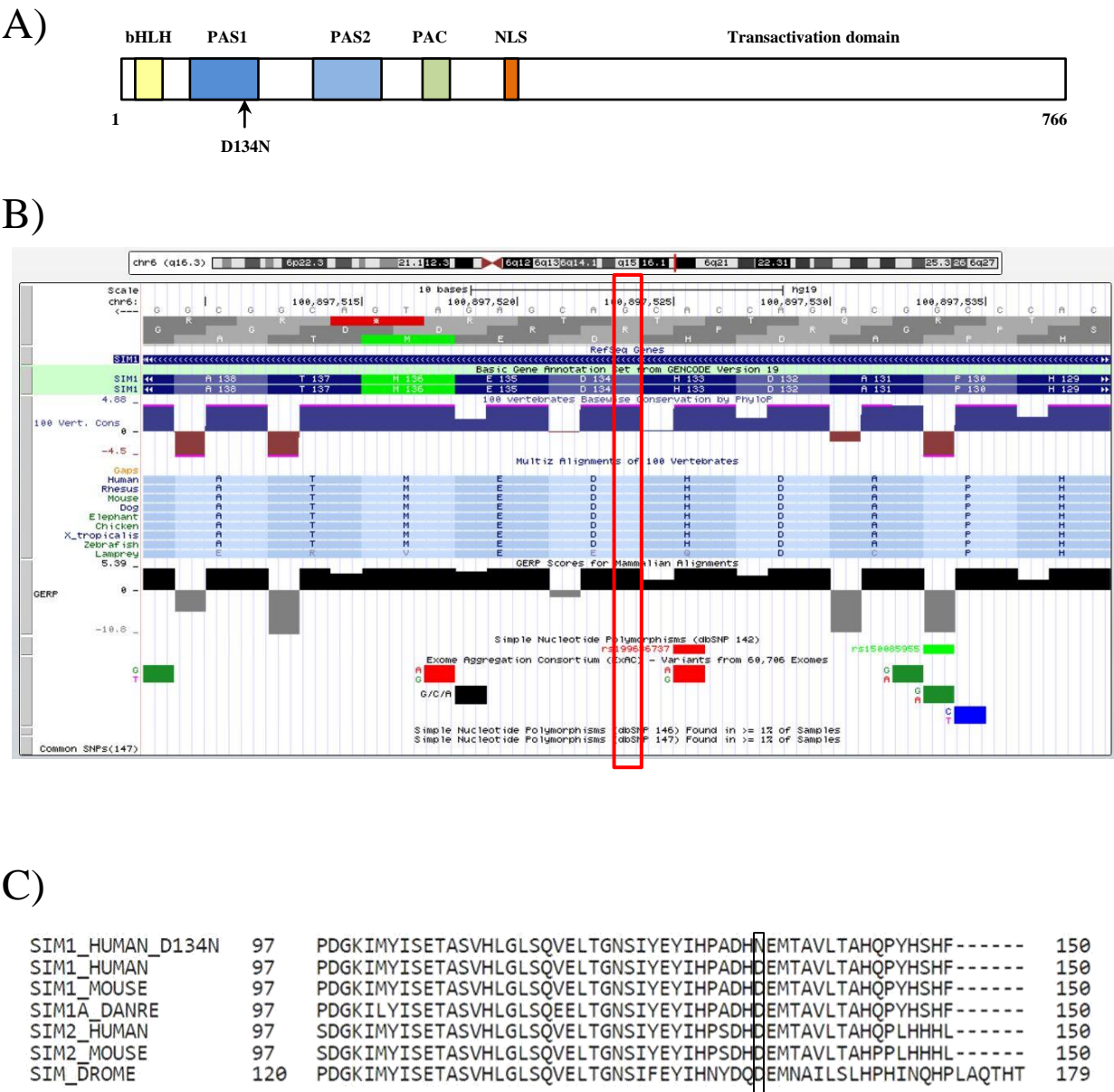

Supplement: S1 Fig — A) The p.D134N is located in the PAS1 domain involved in heterodimerization of SIM1 with ARNT2 transcription factor; B) the aspartic acid at the position 134 is conserved in 99 of 100 vertebrate species as shown in the caption from the UCSC Genome Browser (only one representative species from 8 different vertebrate subsets is displayed); C) The aspartic acid at the position 134 is also conserved in other SIM proteins down to Drosophila melanogaster. (PDF) [file pone.0177222.s001.pdf]

S3 Fig. Indirect calorimetry data.

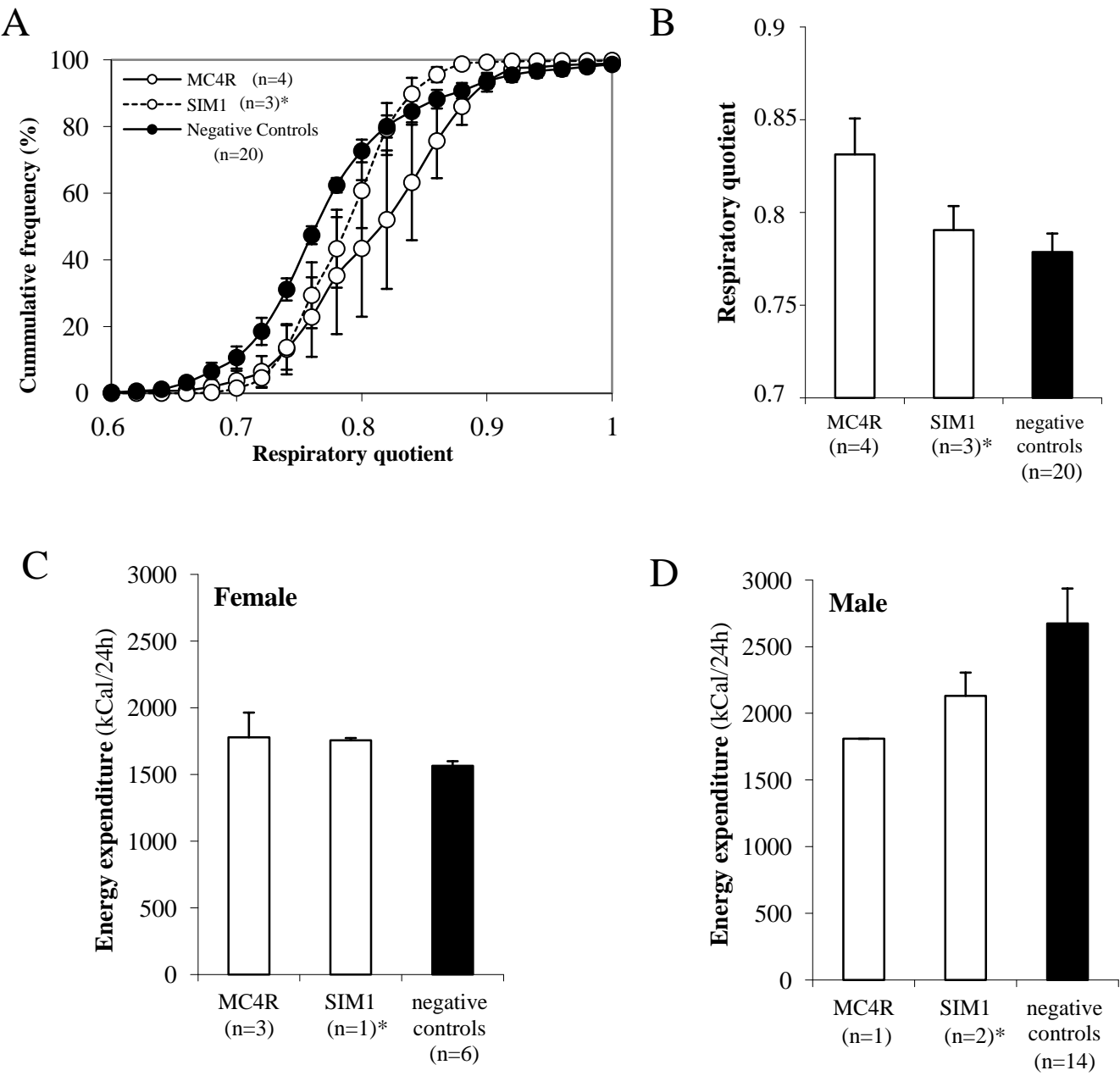

Supplement: S3 Fig — Data (n = 3) are shown as means ± SE. (PDF) [file pone.0177222.s003.pdf]
