# Supplementary material for: Genetic analysis of single-minded 1 gene in early-onset severely obese children and adolescents
Source: PLoS One. 2017 May 4;12(5):e0177222. doi: 10.1371/journal.pone.0177222 (PMC5417716; doi:10.1371/journal.pone.0177222)

S2 Fig. Selected anthropometric and metabolic parameters of the *SIM1* variant p.D134N carriers compared to sex and age matched controls.

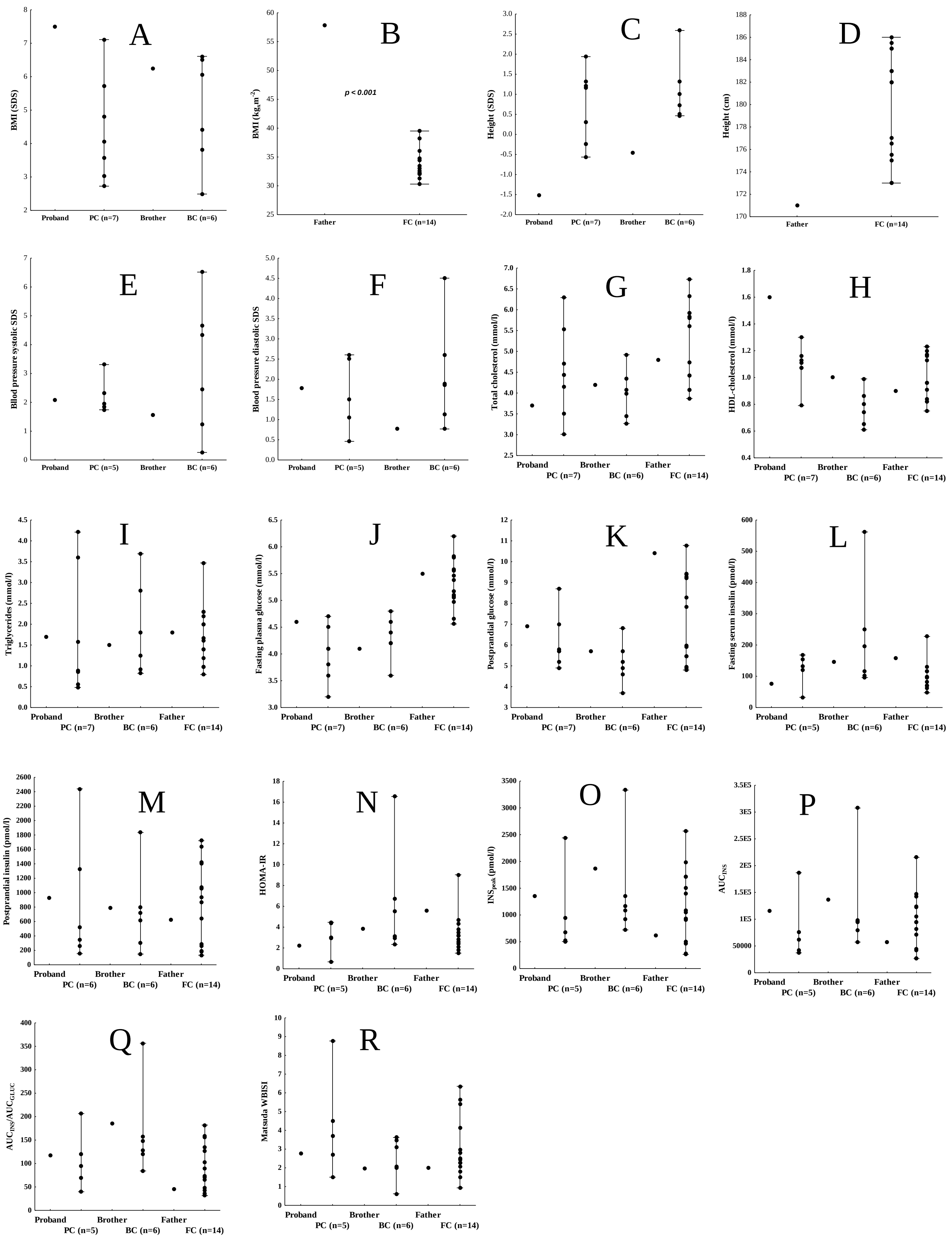

Supplement: S2 Fig — Abbreviations: PC—sex and age matched obese controls for proband, BC—sex and age matched obese controls for proband’s brother, FC—sex and age matched obese controls for proband’s father. The plots display the original values and ranges (for controls). The p-values for differences in unpaired t-test were displayed only if p<0.05. (PDF) [file pone.0177222.s002.pdf]
